# ﻿A new species of a rarely encountered genus *Sclerobregma* Hartman, 1965 (Annelida, Scalibregmatidae) from the deep South China Sea

**DOI:** 10.3897/zookeys.1236.149576

**Published:** 2025-05-02

**Authors:** Jun-Hui Lin, Ya-Qin Huang, Qian-Yong Liang, Xue-Bao He

**Affiliations:** 1 Third Institute of Oceanography, Ministry of Natural Resources, 178 Daxue Road, Xiamen 361005, China Third Institute of Oceanography, Ministry of Natural Resources Xiamen China; 2 National Engineering Research Center of Gas Hydrate Exploration and Development, Guangzhou, 511458, China National Engineering Research Center of Gas Hydrate Exploration and Development Guangzhou China; 3 Guangzhou Marine Geological Survey, China Geological Survey, Guangzhou 511458, China Guangzhou Marine Geological Survey, China Geological Survey Guangzhou China

**Keywords:** Continental slope, deep sea, molecular sequences, morphology, Polychaeta, taxonomy

## Abstract

In the present study, a new species of a rarely encountered genus *Sclerobregma* Hartman, 1965, *Sclerobregmananhaiensis***sp. nov.**, is described based on specimens collected from slope depths of the northern South China Sea. It is characterized by the presence of branched branchiae and heavy acicular spines in the anterior chaetigers, and is morphologically distinct from the other species of the genus in the shape of the anterior margin of the prostomium, the number of neuropodia with nipple-like projections distally, and the segment annulation. Four gene fragments of the new species were sequenced, comprising 16S rRNA, 18S rRNA, 28S rRNA, and histone H3. This study represents the first report of *Sclerobregma* in the South China Sea.

## ﻿Introduction

Annelids of the family Scalibregmatidae are widespread in the world’s oceans, with a broad depth range from the intertidal to the deep sea (Blake 2023). Scalibregmatids mainly feed on organic matter in marine sediment and are generally considered subsurface deposit feeders (Jumars et al. 2015). Compared to many annelid families, Scalibregmatidae is less species-rich, including 88 valid species distributed in 15 genera (Read and Fauchald 2025). The majority of scalibregmatid species prefer to occupy sediment depth greater than 100 m, and knowledge of their diversity in the deep sea is continuously increasing, as evidenced by the numerous species discovered from the lower continental slope or abyssal plains over the past 25 years (Blake 2023), chiefly from the Southern Ocean (Schüller and Hilbig 2007; Schüller 2008), eastern Pacific (Wiklund et al. 2019), southwest Atlantic (Mendes et al. 2023; Mendes et al. 2024a; Mendes et al. 2024b; Mendes et al. 2024c) and off eastern Australian (Blake 2023).

The phylogenetic position of Scalibregmatidae and relationships among its genera remain unclear (Parapar et al. 2021). In a phylogenetic study aiming to resolve the systematic position of *Travisia* (Paul et al. 2010), *Travisia* and scalibregmatids were identified as sister groups to one another, but the inclusion of *Travisia* within Scalibregmatidae was opposed by Blake (2020) due to the great morphological differences. Taxonomically, four scalibregmatid genera possess dorsal and ventral cirri on posterior segments (Blake 2015), i.e., *Oligobregma* Kudenov & Blake, 1978, *Pseudoscalibregma* Ashworth, 1901, *Scalibregma* Rathke, 1843, and *Sclerobregma* Hartman, 1965, and thus are distinguished from the remaining genera. Of these four genera, *Sclerobregma* is unique in that it bears both branched branchiae and heavy acicular spines in anterior chaetigers. The genus *Sclerobregma* was initially erected by Hartman (1965) for the type species *Scl.branchiatum* collected at a depth of 400–2500 m off New England, northwestern Atlantic. The second described species *Sclerobregmastenocerum*, established by Bertelsen and Weston (1980), was later transferred to *Scalibregma* because this species bears short spinous chaetae instead of heavy acicular spines in the anterior body (Mackie 1991), the former chaetae considered to be homologous with lyrate chaetae. Therefore, *Sclerobregma* is a monospecific genus to date. There are few reports of *Sclerobregma* worldwide. Prior to this study, a record of an undescribed species of this genus was reported from the deep eastern Pacific (11.0722°N, 119.655°E); however, it lacked a morphological description (Bonifacio et al. 2020).

The South China Sea (SCS) is the largest semi-enclosed marginal sea in the West Pacific Ocean, with a maximum depth of 5560 m (Wang et al. 2024). Currently, little is known about the diversity of Scalibregmatidae in this region. In the list of annelid species compiled by Glasby et al. (2016), only one scalibregmatid species was recorded, namely *Scalibregmainflatum*, which might be a misidentification based on our findings of molecular identification of the *Scalibregma* specimens from China’s coasts (unpublished data). During several cruises to the continental slope of the northern SCS in recent years, several specimens of *Sclerobregma* were collected from deep-sea sediments. Detailed examination of the available collections determined that they belonged to a new species, which is described and illustrated herein. Four gene fragments were obtained from ethanol-fixed specimen, comprising 16S, 18S, 28S rRNA and histone H3. To the best of our knowledge, this study represents the first report of *Sclerobregma* in the SCS, and even in the Indo-west Pacific.

## ﻿Material and methods

### ﻿Field collection

**Figure 1.**
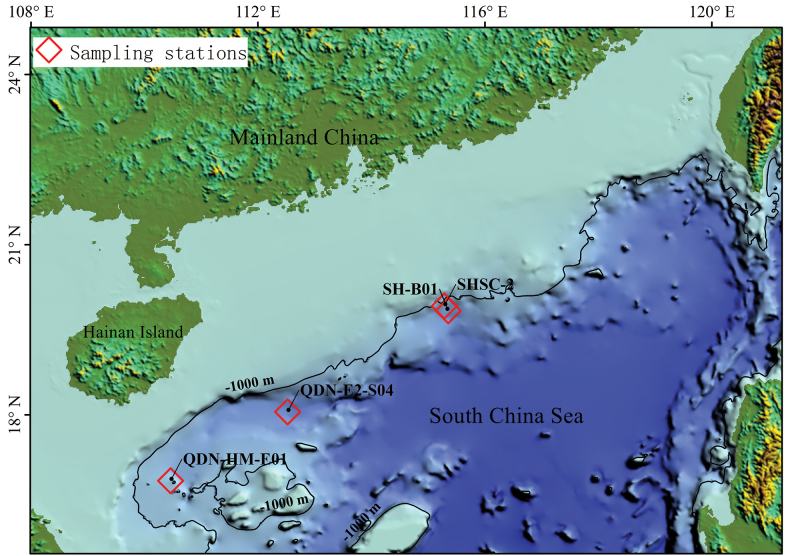
Map of collection localities (squares) of *Sclerobregmananhaiensis* sp. nov.

The specimens examined in this study were collected from the continental slope of the northern South China Sea (Fig. 1) during several cruises organized by the Guangzhou Marine Geological Survey (Guangzhou, China) in recent years. Sediment samples for the analysis of benthic fauna were obtained with a 0.25 m^2^ box corer. Each sample was washed through a 0.25 mm mesh sieve with chilled, filtered seawater (4 °C) on board. The fauna retained by the sieve were fixed in either 95% ethanol or 8% formalin. The tissue of the ethanol-fixed specimen was used for DNA extraction.

### ﻿Morphological observation

In the laboratory, the type and additional specimens were observed using a Leica MZ95 optical stereoscope and a Leica DM6B compound microscope. Parapodia from anterior and posterior segments were dissected and mounted on slides for observation. Light microscope photographs were obtained using a Leica M205A stereoscope equipped with a DFC 550 digital camera. The shape of the chaetae was photographed under a Leica compound microscope (DM6B) with a DFC170 digital camera. SEM observations were not conducted given the paucity of specimens. Plates were prepared using the software Adobe Photoshop CS5. The type material and additional material examined in this study were deposited at the Third Institute of Oceanography, Ministry of Natural Resources, Xiamen, China (**TIO, MNR**).

### ﻿DNA extraction, PCR amplification and sequencing

The total genomic DNA was extracted from the ethanol-fixed specimen using a TransGen Micro Genomic DNA EE 181 Kit (TransGen Biotech, Beijing, China) following the protocol provided by the manufacturer. One mitochondrial (16S) and three nuclear gene markers (18S, 28S, H3) were amplified using primer sets and thermal cycling conditions as delineated by Lin et al. (2023). Then, the 5-μL PCR products were subsequently checked using 1% agarose gel electrophoresis. Sequencing of the successful products was performed in both directions at Sangon (Shanghai, China). Both forward and reverse strands of sequences were manually assembled into a consensus sequence using DNAMAN software (Lynnon Biosoft, Quebec, Canada), then checked for potential contamination using BLAST in GenBank.

### ﻿Data analysis

The partial sequences of the 16S, 18S and 28S rRNA were aligned with those of other *Sclerobregma* species available in GenBank using MAFFT (Katoh et al. 2002) with default settings. The interspecific genetic distances were calculated based on Kimura’s 2-parameter (K2P) model (Kimura 1980) implemented in MEGA X (Kumar et al. 2018).

## ﻿Systematics

### ﻿Class Polychaeta Grube, 1850

**Family Scalibregmatidae Malmgren, 1867**

#### ﻿Genus Sclerobregma

Taxon classificationAnimaliaBoletalesScalibregmatidae

Hartman, 1965

A71388EF-BF49-5725-9A37-9ACC6987039A

##### Type species.

*Sclerobregmabranchiatum* Hartman, 1965.

##### Generic diagnosis.

(after Blake 2020) Body elongate and arenicoliform, ventral groove with elevated pads present from chaetiger 1 along entire body. Prostomium T-shaped with a pair of narrow frontal horns; eyes absent. Peristomium a single inflated ring, encompassing the prostomium dorsally; nuchal organs narrow slits posteriorly between prostomium and peristomium. Parapodia of posterior segments with dorsal and ventral cirri; each cirrus inflated, with numerous internal tubular glands with external openings visible as minute pores using SEM; interramal sense organ present as distinct papilla. Branched pectinate-like branchiae present on chaetigers 2–5. Chaetae include capillaries throughout, lyrate chaetae from chaetiger 3, large acicular spines present on notopodia of chaetigers 1 and 2 and neuropodia of chaetiger 1; few very small blunt-tipped spinous chaetae anterior to acicular spines on chaetigers 1 to 2, representing homologues of lyrate chaetae found on following chaetigers. Pygidium with five anal cirri.

#### ﻿ Sclerobregma nanhaiensis sp. nov.

Taxon classificationAnimaliaBoletalesScalibregmatidae

9E5210D1-53AF-540E-910B-DCC2397F6F7E

https://zoobank.org/22D4D1A4-FB82-4D8A-BB21-526BF49CC7D7

Figs 2A–I , 3A–E

##### Material examined.

***Holotype.*** China • TIO-Poly-149, incomplete; northern South China Sea; sta. 2016SH-B01; 19°50′N, 115°21′E; depth 1601 m; 3 Apr. 2016; Xue-Bao He leg. ***Paratype.*** China • TIO-Poly-150, 1 spec., incomplete; northern South China Sea; sta. 2018SHSC-2; 19°55′N, 115°17′E; depth 1277 m; 11 Apr. 2018; Xue-Bao He leg.

##### Additional material.

China • TIO-Poly-151, 2 specs, incomplete; northern South China Sea; sta. QDN-E2-S04; 18°3′N, 112°31′E; depth 2352 m; 21 Jun. 2019; Jun-Hui Lin leg. • TIO-Poly-152, 1 spec., incomplete; northern South China Sea; sta. QDN-HM-E01; 16°50′N, 110°28′E; depth 1464 m; 3 Jul. 2023; Zhi-Zhong Huang leg.

##### Description.

**Figure 2.**
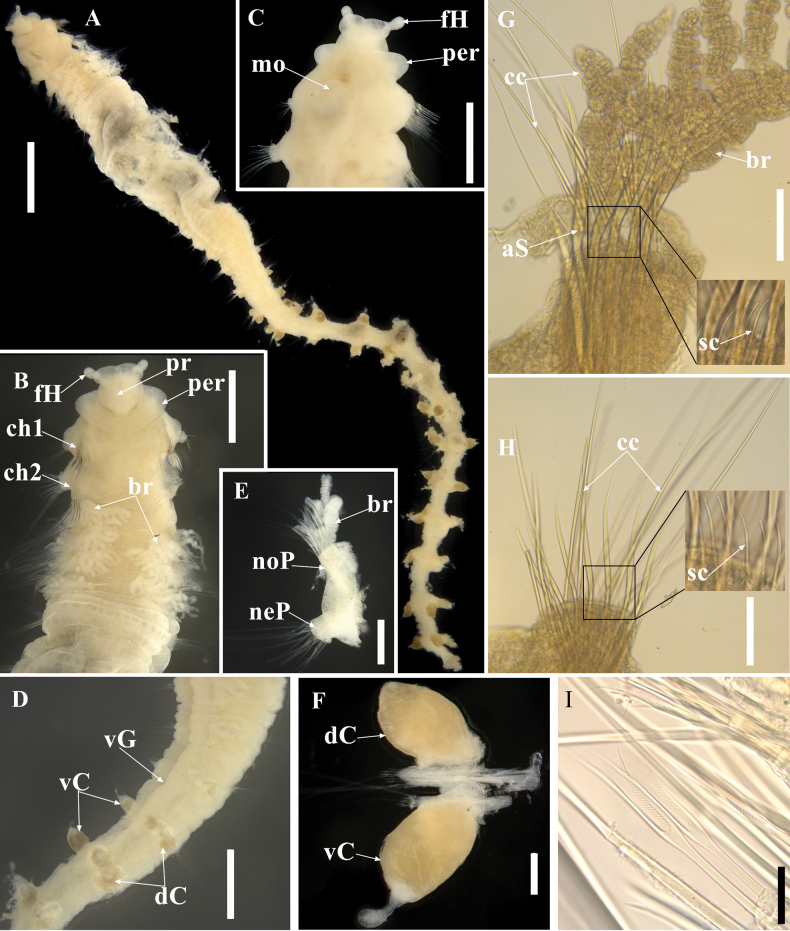
*Sclerobregmananhaiensis* sp. nov. holotype (TIO-Poly-149) **A** entire specimen in dorsal view **B** anterior end in dorsal view (right parapodium of chaetiger 2 dissected) **C** anterior end in ventro-lateral view **D** middle segments in lateral view **E** chaetiger 2 in anterior view **F** chaetiger 28 in anterior view **G** notopodium of chaetiger 2 in anterior view **H** neuropodium of chaetiger 2 in anterior view **I** lyrate chaetae. Abbreviations: aS, acicular spine; br, branchiae; cc, capillary chaetae; ch, chaetiger; dC, dorsal cirrus; fH, frontal horn; mo, mouth; neP, neuropodium; noP, notopodium; per, peristomium; pr, prostomium; sc, spinous chaetae; vC, ventral cirrus; vG, ventral groove. Scale bars: 1 mm (**A**); 500 μm (**B–D**); 200 μm (**E**); 100 μm (**F–H**); 20 μm (**I**).

**Figure 3.**
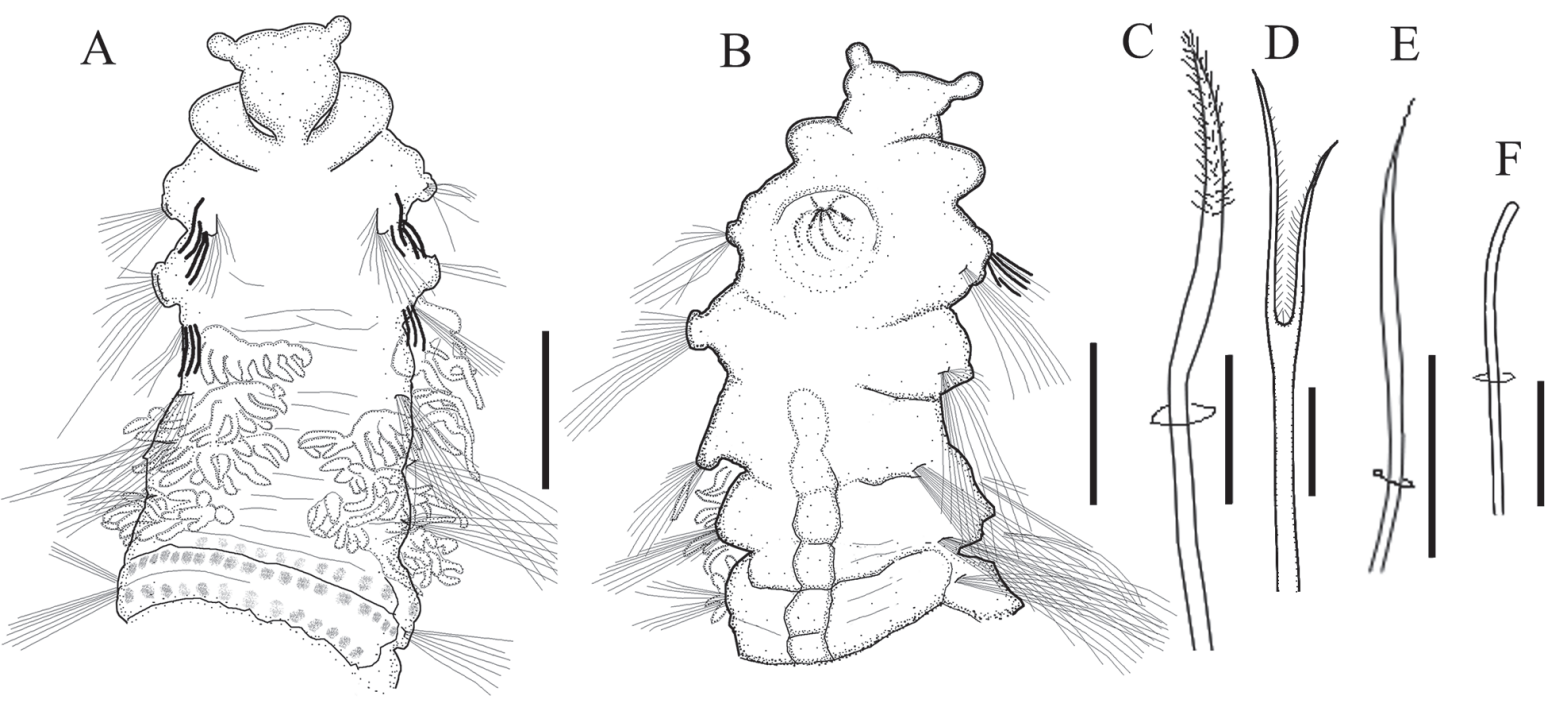
*Sclerobregmananhaiensis* sp. nov. holotype **A** anterior 6 chaetigers in dorsal view **B** anterior 5 chaetigers in ventral view **C** acicular spine on notopodium of chaetiger 2 **D** lyrate chaetae **E** transitionary chaetae from neuropodium of chaetiger 2 **F** spinous chaetae from chaetiger 2. Scale bars: 500 μm (**A, B**); 100 μm (**C, E**); 20 μm (**D**); 25 μm (**F**).

Holotype incomplete, measuring 12.6 mm long by 1.1 mm wide for 29 chaetigers; paratype incomplete, broken into two fragments with 7 chaetigers (2.7 mm long) and 12 chaetigers (4.8 mm long). Body arenicoliform (Fig. 2A), with weakly expanded thoracic region (chaetigers 6–10), narrowing to posterior end. Color in alcohol light tan, without pigmentation. Chaetigers 1–5 smooth (Fig. 2B), without distinct annulation; chaetigers 6–15 quadriannulate; posterior segments with 6 annuli. Chaetigers 6–10 with transverse rows of weakly elevated pads on dorsum (Figs 2A, 3A), closely spaced. Venter with prominent ventral groove bearing elevated pads from chaetiger 3 (Fig. 3B). Four pairs of branched branchiae located on chaetigers 2–5 (Fig. 2B), arising posterior to notochaetae, first branchia smaller than others. Pygidium absent on both specimens.

Prostomium bell-shaped, with anterior margin broadly rounded, medially incised (Fig. 2B, C); posterior part dorsally narrowing into V-shape (Fig. 2B); a pair of nearly spherical frontal horns emerging subapically from anterior margin (Fig. 2B, C), directed laterally or anterolaterally. Eyes absent. Nuchal organs present as slits on posterolateral side of prostomium (Figs 2B, 3A). Proboscis slightly everted in holotype (Fig. 2C). Peristomium a smooth, single-lobed ring around prostomium dorso-laterally, interrupted mid-dorsally, ventrally forming upper lips of mouth. Structure of mouth obscured.

Parapodia biramous with squared parapodial lobes anteriorly (Fig. 2E), becoming conical from midbody to posterior end; parapodial lobes always shorter than cirri. Dorsal and ventral cirri present from chaetiger 14 in holotype, relatively small at first, becoming well developed on subsequent segments, inflated and bright brick-red (Fig. 2D, F). Dorsal cirri ellipsoid, broad basally, tapering to rounded tips; ventral cirri asymmetrical with broad basal attachment and terminated in nipple-like tips. Interramal papillae present between noto- and neuropodia in midbody, obscured in posterior segment.

Heavy recurved acicular spines (Figs 2E, G, 3C) present in notopodia of chaetigers 1 and 2, anterior to capillaries, numbering 9 spines arranged in one row at chaetiger 1 and 6 spines in one row at chaetiger 2; spines tapering to pointed tip bearing arista. Long and thin capillary chaetae (Fig. 2G, H) present in both rami throughout the body. Lyrate chaetae (Figs 2I, 3D) present from chaetiger 3, positioned anteriorly to capillaries, numbering 3–4 per noto- and neuropodium on anterior segments and increasing to 9–10 on posterior ones. Lyrate chaetae short, with unequal tynes bearing short bristles between tynes. A row of 15–20 short, blunt-tipped spinous chaetae per noto- and neuropodia present in chaetigers 1 and 2 (Figs 2G, H, 3F), anterior to all other chaetae. Transitionary chaetae, broader but shorter than capillaries (Fig. 3E), occurred in neuropodia of chaetiger 2.

##### Morphological variations

. Individual variability was observed with respect to the shape of the prostomium and the distribution of branchiae. Specifically, the anterior margin of the prostomium was medially incised in the holotype (TIO-Poly-149) and an additional specimen (TIO-Poly-152), whereas it was truncated in the paratype (TIO-Poly-150). Regarding the branchiae, some may be lost during field collection. The holotype exhibited four complete pairs of branchiae. In contrast, the paratype (TIO-Poly-150) had lost the first branchia on the right side of chaetiger 2.

##### Remarks.

The genus *Sclerobregma* was initially established by Hartman (1965) for the type species *Scl.branchiatum* collected from the northwestern Atlantic, which was characterized by the presence of heavy acicular spines and branched branchiae in anterior chaetigers. Subsequently, Bertelsen and Weston (1980) and Mackie (1991) made some corrections to the original description after careful re-examination of type and additional material. As a result, *Scl.branchiatum* is mainly characterized by the following characters: (1) four pairs of branchiae located on chaetigers 2–5; (2) heavy acicular spines occurring in notopodia of chaetigers 1 and 2 rather than only in chaetiger 1 (also occur in neuropodia of chaetiger 1 as noted by Blake, 2020); (3) posterior segments bearing inflated dorsal and ventral cirri, with nipple-like projections at the tip of the six anteriormost ventral cirri; and (4) short spinous chaetae, homologous with lyrate chaetae, are located on chaetigers 1 and 2, which is not mentioned in the original description by Hartman (1965).

*Sclerobregmananhaiensis* sp. nov. resembles *Scl.branchiatum* with both species sharing many morphological characters, i.e. four pairs of branchiae on chaetigers 2–5, heavy acicular spines in the notopodia of chaetigers 1 and 2, short spinous chaetae in chaetigers 1 and 2, and lyrate chaetae present from chaetiger 3. Besides, they lack eyes on the prostomium. However, both species differ in the following respects:

1. Shape of anterior margin of prostomium. In *Scl.branchiatum*, the anterior margin of the prostomium was broadly curved and smooth, whereas it is medially incised in *Scl.nanhaiensis* sp. nov.;
2. Number of ventral cirri with nipple-like tips on the posterior segments. Nipple-like tips are present in the six anteriormost ventral cirri of *Scl.branchiatum*, while they are present throughout the posterior segments in *Scl.nanhaiensis* sp. nov.;
3. Body annulation. According to the generic diagnosis provided by Blake (2020), a segment of *Scl.branchiatum* possesses up to four annulations along the body while that of *Scl.nanhaiensis* has six annulations on the posterior segments.

##### Etymology.

The specific name “*nanhaiensis*” is derived from Nánhǎi (南海), the Chinese name for the South China Sea, where the specimens were collected.

##### Distribution.

Currently known from the continental slope of the northern South China Sea at water depth between 1277–2352 m.

##### Gene sequences.

In this study, 452 bp of 16S (accession number PV102047), 1665 bp of 18S (PV102048), 976 bp of 28S (PV102046), and 353 bp of H3 (PV102951) were successfully amplified. Currently, there are genetic data available in GenBank for two congeneric species, i.e., *Sclerobregmabranchiatum* (with sequences for 18S rRNA and 28S rRNA) and an undescribed *Sclerobregma* species (with sequences for COI and 16S rRNA). The 18S rRNA and 28S rRNA were highly conserved with the K2P distances of 0% and 0.5% between *Sclerobregmananhaiensis* sp. nov. and *Scl.branchiatum*, respectively. However, the K2P distance was significantly higher for 16S rRNA between the new species and *Sclerobregma* sp.339PB (Accession number MK971015), at 43.4%.

## Supplementary Material

XML Treatment for Sclerobregma

XML Treatment for Sclerobregma nanhaiensis

## ﻿Additional information

### Data availability

All of the data that support the findings of this study are available in the main text.
